# Innovative Cross-Shaped SRC Column–RC Slab Connection: Experimental Investigation and Finite Element Analysis of Punching Shear Behavior

**DOI:** 10.3390/ma18133159

**Published:** 2025-07-03

**Authors:** Wei Zhang, Jianyang Xue, Jinjun Xu, Baoxin Li

**Affiliations:** 1 School of Energy and Architecture, Xihang University, Xi’an 710077, China; xauat_weizhang@163.com; 2 School of Civil Engineering, Xi’an University of Architecture & Technology, Xi’an 710055, China; andenmond@163.com; 3 Key Lab of Structural Engineering and Earthquake Resistance, Ministry of Education (Xi’an University of Architecture and Technology), Xi’an 710055, China; 4 College of Civil Engineering, Nanjing University of Technology, Nanjing 211816, China; jjxu_concrete@njtech.edu.cn

**Keywords:** cross-shaped SRC column, slab–column connection, punching shear capacity, FEA, parametric study

## Abstract

Flat slab structures are extensively utilized in modern construction owing to their efficient load transfer mechanisms and optimized space utilization. Nevertheless, the persistent issue of brittle punching shear failure at connection zones continues to pose significant engineering challenges. This study proposes an innovative cross-shaped steel-reinforced concrete (SRC) column–slab connection. Through combining test and numerical analyses, the failure mechanisms and performance control principles are systematically analyzed. A refined finite element model incorporating material nonlinearity, geometric characteristics, and interface effects is developed, demonstrating less than 3% error upon test validation. Using the validated model, the influence of key parameters—including concrete strength (C30–C60), reinforcement ratio (*ρ =* 0.65–1.77%), shear span–depth ratio (*λ =* 3–6), and limb height-to-thickness ratio (*c*_1_/*c*_2_ = 2–4)—on the punching shear behavior is thoroughly investigated. The results demonstrate that increasing concrete strength synergistically improves both punching shear capacity (by up to 49%) and ductility (by 33%). A critical reinforcement ratio threshold (0.8–1.2%) is identified. When exceeding this range, the punching shear capacity increases by 12%, but reduces ductility by 34%. Additionally, adjusting the shear span–depth ratio enables controlled failure mode transitions and a 24% reduction in punching shear capacity, as well as a 133% increase in displacement capacity. These results offer theoretical support for the design and promotion of this novel structural system.

## 1. Introduction

Flat slab structures are widely adopted in modern construction due to their efficient load transfer and optimal space utilization, offering a highly effective spatial structural system [1]. However, these structures exhibit critical mechanical deficiencies at the connection regions, including insufficient flexural stiffness, weak shear resistance, and poor ductility [2]. Extensive test studies have demonstrated that conventional slab–column connections are prone to brittle punching shear failure under vertical loads, typically characterized by diagonal cracks penetrating the slab and forming a punching shear cone [3]. More critically, this failure mode is sudden and unpredictable; under dynamic loading, it may trigger progressive collapse [4]. Vargas et al. [5] analyzed recent building structural failures and found that approximately 78% of slab–column structural failures were linked to punching shear at the connections, highlighting the urgent need for safer solutions.

To address these challenges, the cross-shaped steel-reinforced concrete (SRC) column–slab system has emerged as a promising structural innovation. Compared to conventional slab–column structures, the cross-shaped SRC system offers two key advantages: (1) its cross-shaped column aligns with architectural walls, enabling integrated column–wall designs for space optimization [6], and (2) the composite action between the steel core and encasing concrete forms a dual lateral-force-resisting system, enhancing structural capacity by approximately 35% [7]. Test results indicate that the displacement ductility coefficient of cross-shaped SRC column–slab systems can reach 5.0, demonstrating exceptional seismic performance [8]. Notably, comparative tests by Lin et al. [9] revealed that the punching shear capacity of cross-shaped SRC connections exceeds that of conventional RC connections by over 45%, with failure modes transitioning from brittle to ductile behavior. These results validate the system’s suitability for high-rise and long-span applications.

Recent studies have highlighted the sensitivity of punching shear performance to key geometric and material parameters such as flange width, slab thickness, and reinforcement detailing. Moreover, interface effects, such as bond-slip between steel and concrete, and localized stress concentrations near the column perimeter have been shown to influence both strength and ductility in composite systems [10,11]. These findings underscore the necessity of refined numerical models and targeted experimental research.

Despite these advances, significant limitations remain in current studies. First, existing design codes exhibit notable deficiencies, particularly the lack of specific provisions for cross-shaped column–slab connections in mainstream design standards such as ACI 318 [12] and GB 50010-2015 [13]. Second, the test database remains insufficient. According to statistics from the slab–column connection database compiled by Shen et al. [14], cross-shaped column specimens account for only about 5% of the 180 slab–column connection tests recorded, while Jia et al. [15] noted that 18 of their 26 cross-shaped column tests used one-way slabs, failing to adequately represent the bidirectional loading conditions encountered in actual structures.

To compensate for the limitations in physical testing, finite element analysis (FEA) has become a vital tool for studying punching behavior. A series of recent investigations have confirmed its ability to simulate failure processes and evaluate the influence of various parameters. For instance, Yang et al. [16] developed a 3D nonlinear model accurately predicting failure processes and the effects of reinforcement ratios and column geometries. Setiawan et al. [17] proposed a simplified method for shear-reinforced flat plates, reliably estimating critical perimeters and load capacity across loading conditions. Choi et al. [18] validated an ABAQUS model with concrete damage plasticity, achieving high precision in failure mode and load–displacement predictions. Wang et al. [19] showed that cross-shaped SRC columns excel in ductility control and failure mode optimization. Collectively, these studies confirm FEA’s dual role in verifying tests and elucidating key performance parameters.

Although the present study focuses on nonlinear structural responses under quasi-static loading, recent developments in time-domain finite element–boundary integral (FE-BI) coupling methods, such as the marching-on-in-degree solver applied in transient electromagnetic analysis by Zhang et al. [20], may provide methodological inspiration for future structural studies involving interface-dominated phenomena and high-fidelity transient behavior. Integrating such advanced computational strategies may help extend current modeling capabilities to broader dynamic scenarios.

Beyond performance, the cross-shaped SRC system also presents practical advantages in construction and cost. Its orthogonal geometry supports standardized formwork and reduces rebar congestion, while allowing the steel core to be prefabricated and rapidly installed. This approach can shorten construction schedules by 20% compared to conventional RC walls. Although steel usage increases material costs by 8–10%, this is offset by reduced labor, thinner slabs, and lower floor heights, resulting in competitive life-cycle costs [21,22].

This study addresses two key research questions through test and numerical investigations: (1) the crack development and failure mechanisms of cross-shaped SRC column–slab connections, and (2) the effects of material properties (concrete strength, reinforcement ratio) and geometric parameters (shear span–depth ratio, limb height-to-thickness ratio) on connection punching shear performance. In particular, the thickness of the slab is regarded as a key geometric factor because it directly affects the effective depth, the critical perimeter, and the stress distribution near the column interface. Two slab thicknesses (120 mm and 150 mm) are selected to reflect the typical range used in actual design and to systematically evaluate the changes in stiffness, ductility and ultimate load. The findings provide fundamental insights for advancing engineering applications of this system.

## 2. Experimental Research

**Figure 1:**
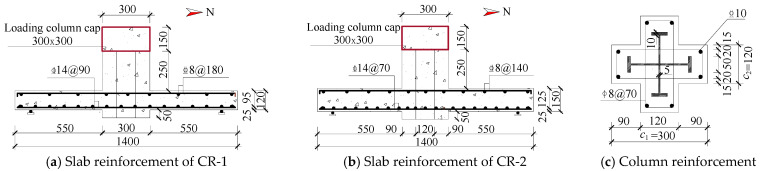
Geometry and reinforcement scheme for the specimens.

As illustrated in Figure 1, two slab specimens were fabricated with dimensions of 1400 × 1400 mm and thicknesses of 120 mm and 150 mm, respectively. Each slab contained bidirectional orthogonal layers of longitudinal steel bars, achieving a designed reinforcement ratio of 1.77%. The SRC cross-shaped columns had a height of 400 mm with a limb height-to-thickness ratio of 2.5. Loading was applied through 300 × 300 mm square stub columns positioned atop the cross-shaped SRC columns at the center of each slab. All concrete elements had a specified strength grade of C30, with concrete cover thicknesses of 10 mm for slabs and 15 mm for columns. Detailed geometric configurations and reinforcement specifications are provided in Figure 1 and Table 1.

### 2.1. Materials Properties

The concrete used in the test slab had a specified 28-day compressive strength of 30 MPa based on standard cube tests, with the actual measured strength reaching 35.6 MPa on the testing day. The structural steel in the columns was Q235B grade. The reinforcement consisted of HRB400-grade steel and HPB300-grade steel, where HRB400 steel was used as longitudinal reinforcement in both the slab (14 mm diameter) and columns (10 mm and 8 mm diameters), while HPB300 steel served as column stirrups (8 mm diameter). Tensile tests showed that the 14 mm, 10 mm, and 8 mm diameter rebars had yield strengths of 436 MPa, 462 MPa, and 445 MPa, respectively, and an elastic modulus of 200,000 MPa.

### 2.2. Testing Device

**Figure 2:**
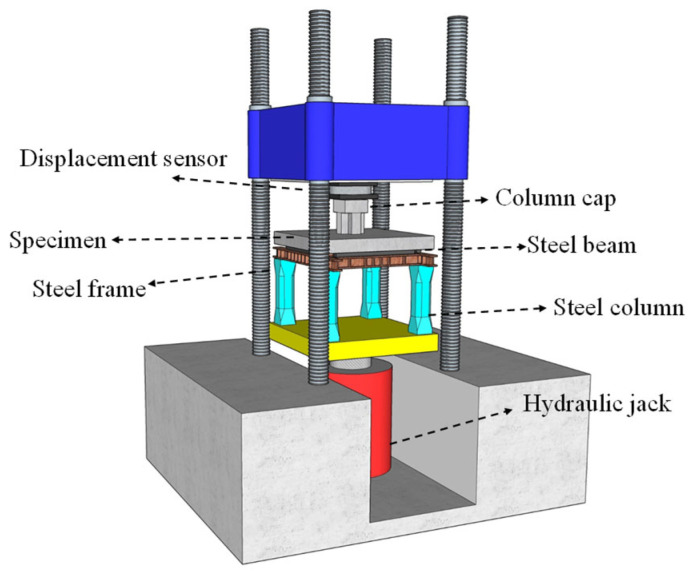
Loading schematic diagram.

During the test preparation phase, the specimen slab was positioned bottom-face-down on a horizontal steel beam system comprising four welded I-beams, which were supported by four equally heighted steel pedestals to create observation space for bottom-face crack development. Four cylindrical rollers serving as supports were placed on the steel beams and symmetrically arranged about the slab center with their axes located 100 mm from the slab edges. These rollers were rotationally free but laterally constrained to prevent horizontal displacement. The loading column head surface was leveled with fine sand and covered with a steel bearing plate of 15 mm thickness to ensure uniform load distribution. A load cell was installed atop this bearing plate, followed by another steel plate interfacing with the testing machine. For monitoring purposes, fisheye cameras were strategically positioned near the column face at both the top and bottom surfaces of the slab to record the joint failure progression. The specimen slab was simply supported on a rigid steel frame while being subjected to concentric loading applied in the displacement control mode at 0.2 mm/s until failure. The load was transferred through a 300 mm × 300 mm column cap using a 2000-ton compression-shear testing machine (YAJ-20000 (Changchun Testing Machine Research Institute, Changchun, China)), as illustrated in Figure 2.

### 2.3. Measurement Scheme

**Figure 3:**
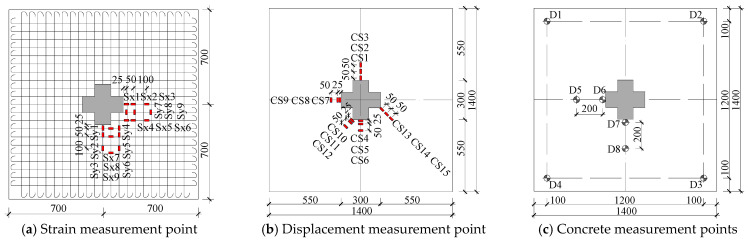
Detailed layout drawing of measurement points.

The displacement and strain data from all measurement points were collected using a TDS-530 static data acquisition system. Steel reinforcement strains were measured using BX-120-5AA strain gauges (Tokyo Sokki Kenkyujo Co., Ltd., Tokyo, China) installed on rebars within approximately 200 mm from the column face, arranged in both x and y directions (Figure 3a). For concrete strain measurements, BX-120-50AA gauges were deployed within a 150 mm radius from the column head on the slab surface, specifically targeting three critical regions: outside the column limbs, at the column re-entrant corners, and outside the column salient corners, with the concrete strain gauge layout detailed in Figure 3b. Displacement measurements were obtained using resistive displacement transducers (YHD-50 and YHD-100 (Nanjing Geotechnical Instruments Co., Ltd., Nanjing, China)) positioned at the column head and surrounding slab locations, as illustrated in Figure 3c. Throughout the test, crack propagation was closely monitored and all cracks were systematically recorded and marked for documentation.

## 3. Finite Element Simulations

### 3.1. Material Constitutive Model

**Figure 4:**
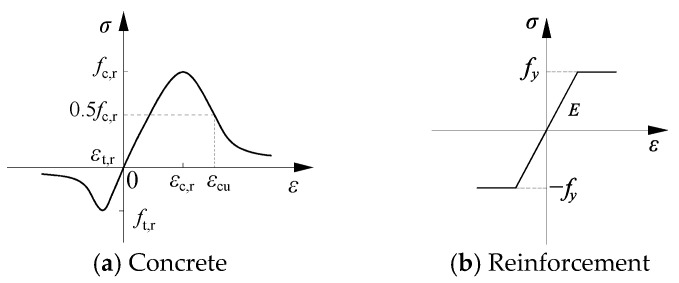
Stress–strain relationship for material.

The mechanical behavior of concrete was simulated using the concrete damaged plasticity (CDP) model in ABAQUS 6.14, which is based on a continuum mechanics framework and effectively captures damage evolution under cyclic loading, making it particularly suitable for analyzing slab–column connections under vertical loads. The compressive stress–strain relationship was defined according to the piecewise curve equation specified in the Chinese design code GB50010-2010 [23] (Figure 4a). The CDP model parameters consist of elastic and plastic components, with additional parameters defining the flow potential, yield surface, and viscosity. The yield function incorporates several key parameters: the biaxial-to-uniaxial compressive strength ratio (*σ*_b1_/*σ*_b2_ = 1.16) and the ratio of second stress invariant to tensile/compressive meridians (*K*_c_ = 2/3), the latter being a commonly adopted value in the literature. The flow potential function is characterized by plastic flow eccentricity (*e* = 0.1) and dilation angle (*ψ*), which was varied between 30° and 42° to investigate its influence on simulation accuracy. Comparative analyses revealed that *ψ* = 38° best replicated the experimentally observed behavior. The viscosity parameter (*v* = 0.001), a time-dependent factor critical for convergence and computational accuracy, was determined through parametric studies. Full parameter details are listed in Table 2.

For reinforcement and structural steel components, a bilinear elastic–plastic constitutive model with isotropic hardening was employed (Figure 4b), where the yield strength was set to the standard value *f*_y_ and the hardening modulus was taken as 0.01 times the initial elastic modulus to simulate post-yield behavior.

### 3.2. Unit Type and Grid Division

**Figure 5:**
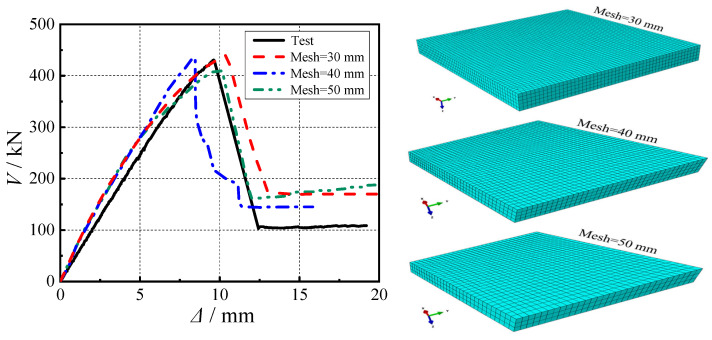
Load–deflection curves for FEA with different mesh sizes.

Through convergence analysis, different element types were carefully selected for refined modeling of concrete, reinforcement, and structural steel components: eight-node linear brick elements (C3D8R) for concrete, two-node linear truss elements (T3D2) for reinforcement, and quadrilateral reduced-integration shell elements (S4R) for structural steel members. The simulation accuracy critically depended on mesh refinement. To optimize computational efficiency while maintaining accuracy, a systematic mesh sensitivity study was conducted comparing three element sizes (30 mm, 40 mm, and 50 mm), as shown in Figure 5. Results showed that the predicted punching shear capacity became stable when the mesh size was reduced below 40 mm, indicating that finer meshes significantly enhanced the model’s ability to capture critical load responses.

Based on these findings, a zoned meshing strategy was implemented: a fine 30 mm mesh was adopted in critical regions (e.g., connection core zones) to ensure accuracy, while coarser meshes were applied in less critical areas. For modeling consistency, both structural steel and reinforcement employed a uniform 50 mm mesh. This optimized approach not only reduced computational costs and mitigated numerical convergence issues, but also reliably simulated the initiation and propagation of diagonal cracks at the slab bottom, thereby providing a dependable basis for structural performance analysis.

### 3.3. Interaction

The numerical simulation adopted appropriate contact and constraint conditions to accurately capture the interaction between components. For the concrete–steel column interface, a Coulomb friction model with a coefficient of *μ* = 0.6 was applied, allowing for shear transfer up to a critical limit dependent on the normal compressive stress. To simulate the interaction between reinforcement, structural steel, and surrounding concrete, a surface-based cohesive contact model was employed. This model accounts for the bond-slip behavior by incorporating normal and tangential stiffness parameters, allowing partial slip and gradual degradation of bond strength after cracking. Unlike the Embedded Region method, which enforces perfect displacement compatibility, the cohesive contact approach enables relative movement at the interface, more realistically capturing the post-peak mechanical response. Although this method slightly increases computational costs, it significantly enhances the accuracy of simulating interface degradation. While accurately capturing peak load behavior, it also better reflects the post-cracking load transfer degradation and improves post-peak predictions.

### 3.4. Boundary Conditions and Loading

The finite element model employed displacement-controlled loading through a reference point connected to the loading column’s upper surface via multi-point coupling constraints. Boundary conditions were carefully designed to replicate experimental conditions: the specimen was allowed free deformation in the XY plane while maintaining geometric stability through simply supported conditions with one fixed and one pinned support. This configuration accurately simulated the actual test setup while preventing rigid body displacement. Additionally, vertical constraints were applied along the supported edges to properly represent the boundary behavior.

The loading protocol consisted of three distinct phases: (1) elastic phase (pre-cracking), with 0.2 mm displacement increments; (2) post-cracking phase, using 0.4 mm increments for computational efficiency; and (3) strengthening phase (beyond 80% of peak load *P*_u_); this is followed by reverting to 0.2 mm increments to ensure convergence accuracy until the load dropped to 65% of *P*_u_. A reference node at the slab center, coupled to the loading column surface, ensured uniform displacement distribution, effectively simulating the quasi-static loading applied by hydraulic jacks in physical tests. This incremental loading strategy successfully balanced numerical stability with accurate representation of nonlinear structural responses.

## 4. Comparison of Finite Element and Test Results

### 4.1. Failure Mode and Cracking Mode

**Figure 6:**
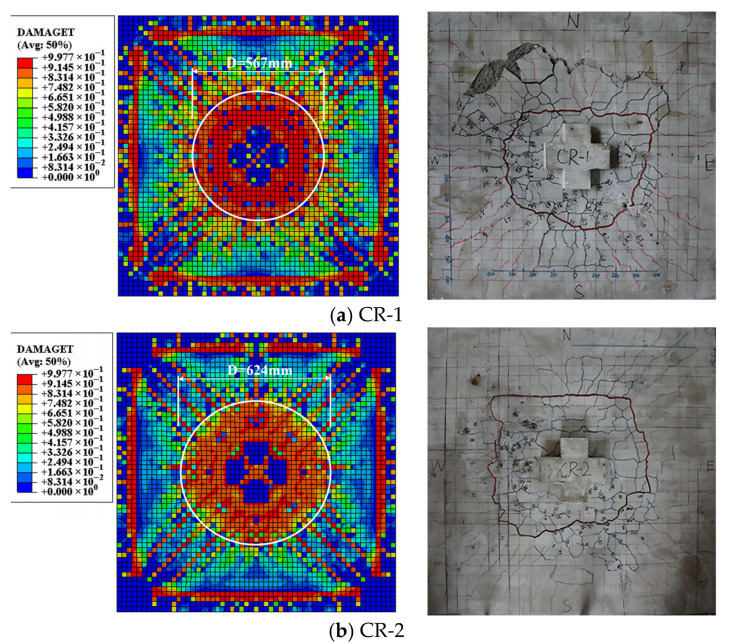
Test and FEA cracking pattern at failure of the specimens.

Both specimens CR-1 and CR-2 exhibited typical brittle punching shear failure modes, with their failure processes developing through four distinct stages: (1) initial radial cracks emerging at column corners, (2) flexural cracks appearing in the central slab region and propagating toward edges, (3) gradual formation and extension of circumferential cracks with increasing load, and (4) sudden load-bearing capacity loss when circumferential cracks interconnected. Test observations showed good agreement with finite element analysis (FEA) results regarding crack propagation patterns. However, CR-2 (with increased slab thickness and consequently higher stiffness) demonstrated fewer cracks with reduced widths and no concrete spalling, as illustrated in Figure 6b. Through post-test cleaning of loose concrete debris and tracing the circumferential cracks with maximum depth, it was observed that the actual punching shear failure surface exhibited an irregular (approximately circular) shape. This finding differs from the rectangular column punching failure surface morphologies reported by Oleiwi [24] and Jia & Luin [25], demonstrating that column cross-sectional geometry significantly influences the geometric characteristics of failure surfaces.

Furthermore, in the finite element analysis, the maximum orthogonal crack envelope region was simplified as a circular failure surface for calculation based on the isotropic material assumption. The white area shown in Figure 6 represents the calculated failure zone using the equivalent circle method. Results in Table 3 demonstrate good agreement between experimental and test simulations regarding both the morphology and dimensions of the failure surface. Significantly, both approaches consistently confirmed that increasing slab thickness leads to a slight expansion of the failure perimeter radius, which consequently enlarges the punching shear failure surface area and ultimately enhances the specimen’s ultimate bearing capacity. This finding provides crucial evidence for understanding the influence of slab thickness on punching shear performance.

### 4.2. Comparative Analysis of Load–Displacement Curves

**Figure 7:**
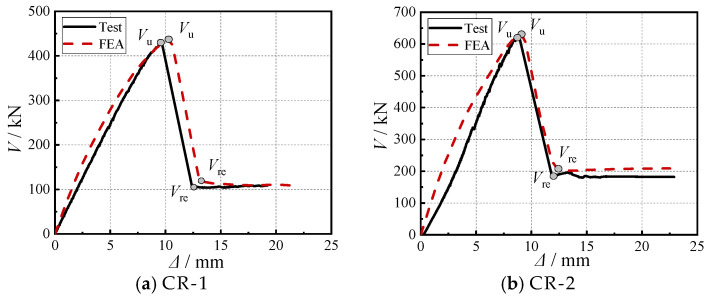
Comparison of load–displacement curves between test and FEA results.

Figure 7 presents the load–displacement curves comparing test and FEA results, demonstrating excellent overall agreement with both exhibiting typical brittle failure characteristics. During initial loading, the test and numerical initial stiffness showed over 95% consistency, validating model reliability. As loading progressed, the test curve maintained near-linear behavior while the FEA results displayed more pronounced stiffness degradation. This discrepancy primarily stems from two factors: (1) the concrete in the test slab developing microcracks due to casting processes, temperature variations, or shrinkage effects, all of which contributed to the reduction in the slab’s initial stiffness; and (2) the finite element model’s ideal elastoplastic constitutive relationship, which more sensitively captured material nonlinearity. Notably, specimens with greater slab thickness exhibited higher initial stiffness due to increased moment of inertia.

The test and finite element analysis results showed good agreement, with a discrepancy of less than 3% in ultimate load capacity (*V*_u_), as presented in Table 4, confirming the model’s validity. The residual load (*V*_re_) also exhibited a deviation of less than 13%. Although this difference is slightly larger than that for *V*_u_, the use of a surface-based cohesive contact model to represent the bond–slip interaction between steel and concrete effectively captured the key mechanical behaviors observed in the tests, including concrete cracking patterns and stress development. The model also demonstrated particularly high accuracy in predicting the pre-peak load response. These results indicate that the developed finite element model reliably simulates the punching shear behavior of cross-shaped SRC column–slab connections and provides a sound theoretical basis for subsequent parametric studies.

## 5. Parameter Analysis

### 5.1. Parameter Setting

The punching shear performance of cross-shaped SRC column–slab connections is governed by multiple inter-related parameters, which can be categorized into three groups: material parameters (e.g., concrete strength, steel strength), geometric parameters (e.g., slab thickness, column cross-section dimensions, shear span–depth ratio), and structural parameters (e.g., reinforcement ratio, stirrup configuration). To address the limitations of test studies in parameter coverage and cost-effectiveness, this study utilizes a test-validated ABAQUS finite element model to conduct a comprehensive parametric analysis. Based on sensitivity analysis and engineering applicability considerations, four key parameters are investigated: (1) concrete compressive strength (30–60 MPa), which governs punching shear capacity and crack propagation characteristics; (2) flexural reinforcement ratio (*ρ =* 0.65–1.77%), dominating flexural stiffness and ductile behavior; (3) shear span–depth ratio (*λ =* 3–6), controlling the transition between punching shear and flexural failure modes; and (4) limb height-to-thickness ratio (*c*_1_/*c*_2_ = 2–4), influencing stiffness distribution and stress concentration in column limbs, thereby altering shear transfer mechanisms.

The parametric study adopts a controlled variable approach, with common design values used as reference points (see Table 5), to systematically investigate the individual effects of various parameters on the connection’s punching shear capacity, stiffness degradation patterns, and crack development modes. By establishing quantitative relationships between parameters and performance indicators, the research revealed the regulatory mechanisms of different parameters on the punching shear behavior of connections, providing a scientific basis for optimal connection design. Particularly, in-depth analysis was conducted on the critical thresholds of parameters and their synergistic effects, offering guidance for determining appropriate values of key parameters in engineering practice.

### 5.2. Concrete Strength

**Figure 8:**
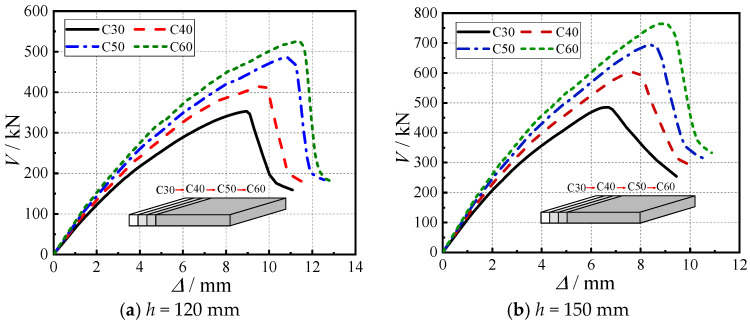
Load–displacement curves for different concrete strengths.

By establishing finite element models with four concrete strength grades (C30–C60), this study systematically analyzed the influence of concrete strength on the punching shear behavior of cross-shaped SRC column–slab connections, as illustrated in Figure 8. The results demonstrate that all specimens (including both 120 mm and 150 mm slab thicknesses) exhibited typical punching shear failure characteristics, manifested by severe concrete damage around the column head and distinct brittle failure patterns. Taking the 150 mm thick models as an example, when concrete strength increased from C30 to C60, the connection’s initial stiffness and unloading stiffness improved by approximately 18–22%, and the ultimate load capacity showed a cumulative increase of 49%, with incremental gains of 24% from C30 to C40, 15% from C40 to C50, and 10% from C50 to C60. The peak displacement accumulated a 33% increase, and the ductility coefficient rose from 2.1 to 2.8, representing a total improvement of 33%. These mechanical improvements primarily result from high-strength concrete’s superior crack resistance, enhanced steel–concrete composite action, and more uniform connection zone stress distribution. The findings confirm that appropriately increasing concrete strength in engineering practice can simultaneously enhance both the punching shear capacity and ductility performance of the connections, providing an effective technical measure for improving the punching shear behavior of cross-shaped SRC column–slab connections.

### 5.3. Flexural Reinforcement Ratio

**Figure 9:**
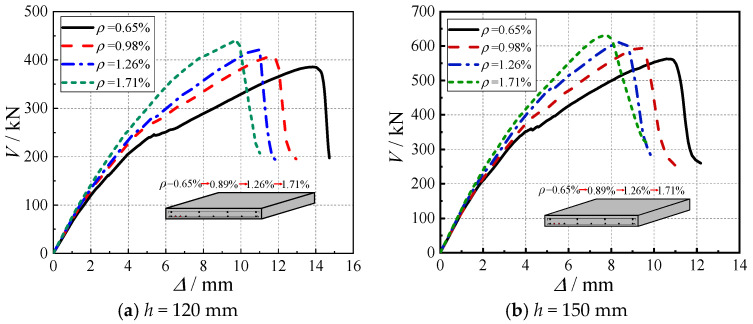
Load–displacement curves under different reinforcement ratios.

A series of finite element models with reinforcement ratios (*ρ*) ranging from 0.65% to 1.77% were established to investigate the influence of flexural reinforcement on the punching shear behavior of cross-shaped SRC column–RC slab connections, as shown in Figure 9. The numerical results demonstrated that all models exhibited typical punching shear failure characteristics, including the following: (1) sudden strength degradation after peak load, (2) formation of penetrating diagonal cracks in concrete, and (3) limited yielding of flexural reinforcement. For the 150 mm thickness models, increasing the reinforcement ratio from 0.65% to 1.77% resulted in the following mechanical responses: (1) the ultimate load capacity (*V*_u_) increased by only 12% with incremental gains of 6%, 3%, and 3%, indicating diminishing returns; (2) the peak displacement (Δ_u_) decreased substantially by 30% with reduction rates of 13%, 11%, and 9%; and (3) the ductility coefficient (Δ_u_/Δ_y_) decreased from 3.2 to 2.1, representing a 34% reduction. These mechanical performance variations were governed by three primary mechanisms: first, the bond-slip effect induced by concrete cracking weakened the anchorage performance; second, the anchorage zones in high-reinforcement-ratio specimens were severely damaged during punching shear failure; third, specimens with lower reinforcement ratios exhibited more pronounced yield characteristics due to better stress redistribution capacity.

Based on the parametric sensitivity analysis, an optimal reinforcement ratio range of 0.8–1.2% is recommended for engineering design. This range ensures a load capacity improvement within 15% while limiting ductility deterioration to an acceptable level (<25%), thereby achieving a balanced optimization between bearing capacity and ductility performance for the cross-shaped SRC column–RC slab connections.

### 5.4. Shear Span–Depth Ratio

**Figure 10:**
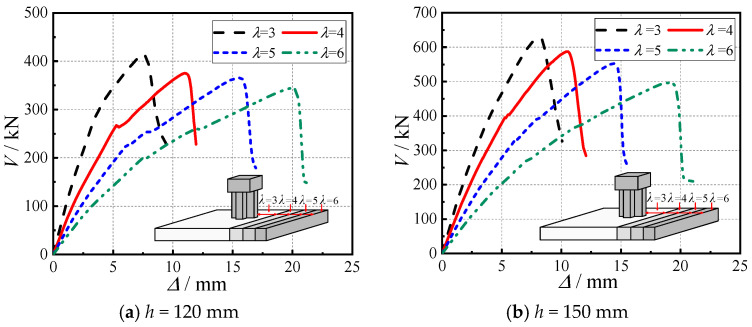
Load–displacement curves under different shear span–depth ratios.

To address the discrepancy between the small shear span–depth ratio (resulting in brittle punching shear failures) observed in laboratory tests due to equipment limitations and the larger ratios typically encountered in practical engineering, finite element models with two slab thicknesses (120 mm and 150 mm) were developed to analyze the influence mechanism of shear span–depth ratios (*λ* = 3–6) on the punching shear behavior of SRC cross-shaped column–RC slab connections, as summarized in Figure 10. Comparative results revealed that while all models with different shear span–depth ratios ultimately failed in punching shear, the failure displacement increased significantly with higher *λ* values. For 150 mm thickness models, when the shear–span ratio increased from 3 to 6, the displacement enhancement grew from 27% to 133%. Conversely, the ultimate load capacity showed a decreasing trend, a 24% cumulative reduction was observed for the 150 mm model as *λ* increased from 3 to 6, accompanied by marked ductility improvement (ductility coefficient increased from 1.96 to 2.60).

This behavioral transition fundamentally stems from the increased horizontal distance between concentrated load and slab edge at higher shear–span ratios, which raises the ratio of the flexural bearing capacity to the impact shear-bearing capacity of the connection, promoting a failure mode transition from pure punching toward combined flexure–shear failure. This manifests as greater failure displacement and improved ductility with more pronounced flexural failure features. Although the fundamental failure mode remains unchanged, the substantially enhanced ductility makes the failure process more predictable. The findings elucidate the intrinsic mechanism of shear span–depth ratio’s influence on failure mode transition, suggesting that in engineering practice, failure modes and ductility performance can be effectively regulated through deliberate adjustment of the shear span–depth ratio.

### 5.5. Limb Height-to-Thickness Ratio

**Figure 11:**
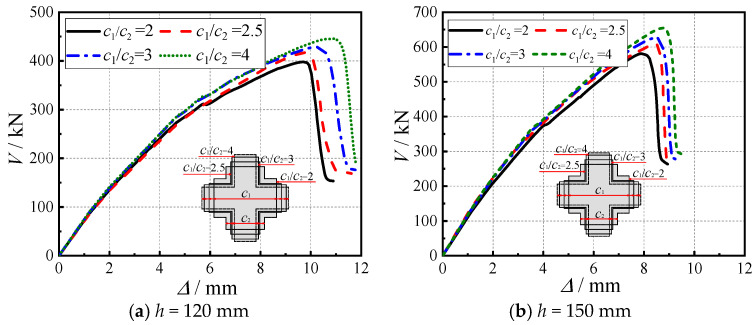
Load–displacement curves under different limb height-to-thickness ratios.

Under the condition of maintaining constant column cross-sectional area, this study systematically investigated the influence mechanism of limb height-to-thickness ratio (*c*_1_/*c*_2_ = 2–4) on the punching shear behavior of SRC cross-shaped column–RC slab connections, as illustrated in Figure 11. The finite element analysis results demonstrate that when *c*_1_/*c*_2_ increased from 2 to 4, the 150 mm thickness slab models exhibited a 14% cumulative improvement in ultimate load capacity (with incremental increases of 7%, 3%, and 4%) and a corresponding 6% increase in displacement (with increments of 1%, 2%, and 3%). This performance enhancement primarily originates from the increased effective sectional area caused by column shape modification, resulting in an approximately linear growth relationship between critical perimeter and punching shear capacity. However, when *c*_1_/*c*_2_ exceeds a critical threshold, the intensified stress concentration effects induced by geometric discontinuities in column limbs lead to uneven shear stress distribution, potentially compromising the connection’s punching shear capacity.

The results indicate that modifying the height-to-thickness ratio of the connection effectively alters the effective load-bearing area and the internal stress distribution, thereby regulating the punching shear capacity. Both the load-bearing capacity and ductility exhibit a relatively obvious sensitivity to variations in limb width or aspect ratio. For engineering design, it is recommended to balance the dual considerations of ultimate bearing capacity and stress concentration effects by maintaining *c*_1_/*c*_2_ within an optimal range to achieve peak performance.

## 6. Comparison with Code Predictions

Structural design codes offer design expressions for estimating the punching shear resistance of slabs such as those provided by GB 50010-2015, ACI 318, and Eurocode 2 [26], summarized in Table 6.

Based on the crack propagation patterns and failure modes observed on the slab underside in Section 4.1, the analysis indicates that the punching shear damage in cross-shaped column–slab connections is primarily concentrated within a radial distance of approximately 0.8*h*_0_ to 1.5*h*_0_ from the column corners. Given the limitations of current design codes in addressing the critical perimeter for non-rectangular columns, this study proposes an improved critical perimeter assumption tailored for cross-shaped column–slab connections. Detailed parameter recommendations are provided in Table 7. This revised assumption accounts for the distinct stress distribution characteristics observed in cross-shaped column–slab connections and offers enhanced applicability and reference value compared to conventional square column assumptions, thereby strengthening the theoretical basis for slab systems with non-standard column geometries.

To systematically evaluate the accuracy of existing design codes in predicting the punching shear capacity of cross-shaped column–slab connections, code-based predictions were comprehensively compared with test and finite element analysis (FEA) results. The evaluation framework includes the following: (1) design equations from major international codes, as summarized in Table 8; and (2) critical perimeter definitions corresponding to each design method, illustrated in Table 7.

As shown in Table 8, FEA predictions exhibited the highest accuracy, with an average *V*_Test_/*V*_c_ ratio of 0.99. In contrast, predictions from GB50010-2015, ACI 318, and Eurocode 2 yielded average ratios of 1.86, 2.00, and 1.50, respectively. Although all three codes provide conservative estimates relative to experimental values, Eurocode 2 demonstrates comparatively better accuracy. This discrepancy is largely attributed to the assumed critical perimeter; GB50010-2015 and ACI 318 adopt a perimeter at 0.5*h*_0_ from the column face, while Eurocode 2 uses a value of 2*h*_0_, which more effectively mitigates stress concentration at column corners. The 0.5*h*_0_ perimeter fails to account for this localized stress field adequately.

However, despite 2*h*_0_ showing better agreement with test results, none of the existing code provisions fully reflect the actual deformation and failure mechanisms observed in cross-shaped column–slab connections. Thus, while the general trends of predictions are consistent, there remains room for improving accuracy.

Furthermore, although the FEA approach provides the most precise and least variable predictions, it is recommended that a reduction factor be introduced to account for the influence of column cross-sectional shape. This would be similar in spirit to the strength reduction factor used in ACI 318 for rectangular columns, helping to account for the reduced punching resistance caused by irregular column geometries.

## 7. Conclusions

Through test investigations and ABAQUS parametric analyses, this study systematically examines the punching shear behavior of SRC cross-shaped column–RC slab connections, yielding the following key findings:

(1) The connections exhibit characteristic punching shear failure mechanisms: initial radial tensile cracks develop at the slab bottom, followed by progressive tensile damage propagation along 45° inclined planes toward the top surface, concurrent with compressive damage evolution from the slab mid-depth upward. Catastrophic punching failure occurs once tensile damage reaches the top surface and the compressive concrete attains its ultimate strain.
(2) Increasing concrete strength (C30 to C60) significantly enhances connection performance, yielding a 49% cumulative increase in ultimate capacity, a 33% increase in ductility coefficient, and an 18–22% improvement in stiffness. These benefits originate from improved crack resistance, optimized steel–concrete composite action, and more uniform stress distribution, providing scientific justification for selecting higher concrete grades in engineering applications.
(3) The reinforcement ratio (*ρ =* 0.65–1.77%) demonstrates only marginal effects on punching shear capacity (maximum 12% increase) but substantially reduces ductility (34% deterioration). An optimal range of 0.8–1.2% is recommended to balance strength and ductility, while avoiding anchorage performance degradation and loss of stress redistribution capacity associated with higher reinforcement ratios.
(4) Larger shear span–depth ratios (*λ* = 3–6) reduce ultimate capacity (24% reduction) while remarkably enhancing displacement capacity (133% increase) and ductility (33% higher coefficient). This behavior reflects a transition from brittle punching shear to a combined flexure–shear failure mode, driven by changes in moment-to-shear capacity ratios, offering valuable insights for ductile connection design.
(5) Under constant cross-sectional area conditions, increasing the limb height-to-thickness ratio (*c*_1_/*c*_2_ = 2–4) improves both the ultimate load capacity (with a cumulative increase of 14%) and displacement capability (6%), with these effects being governed by the dual mechanisms of effective calculated sectional area and stress concentration. However, excessively high height-to-thickness ratios may induce stress concentration effects due to geometric discontinuities in column limbs, thereby diminishing the performance gains.

## Figures and Tables

**Table 1:** Design parameters of specimens.

Specimens	Limb Height	Limb Thickness	Reinforcement Ratio	Slab Thickness
	*c*_1_ (mm)	*c*_2_ (mm)	*ρ* (%)	*h* (mm)
CR-1	300	120	1.77	120
CR-2	300	120	1.77	150

**Table 2:** Concrete parameters.

*f*_cu_/MPa	*E*_c_	*u*	*ψ*	*e*	*σ*_b1_/*σ*_b2_	*K*_c_	*v*
35.6	31498.8	0.2	38	0.1	1.16	2/3	0.00001

**Table 3:** Comparison of the punching shear failure location of the specimens.

Specimens	Failure Area *A*/mm^2^		Failure Perimeter *L*_p_/mm	
	Test	FEA	Test	FEA
CR-1	245,280	252,368	1667	1780
CR-2	290,211	305,660	1854	1960

**Table 4:** FEA and test punching shear capacity.

Specimens	Ultimate Load *V*_u_/kN		Residual Load *V*_re_/kN		*V*_u_	*V*_re_
	Test	FEA	Test	FEA	*V*_Test_/*V*_FEA_	*V*_Test_/*V*_FEA_
CR-1	430.75	439.66	109.37	122.47	0.98	0.89
CR-2	625.64	630.34	185.88	209.13	0.99	0.89

**Table 5:** Design parameters.

Design Parameters	Specimens	Grades	*h*/mm		*l*_0_/mm	*ρ*/%	*c*_1_/*c*_2_
Concrete strength	C30	C30	120	150	1200	1.77	2.5
	C40	C40					
	C50	C50					
	C60	C60					
Reinforcement ratio	*ρ =* 0.65%	C40	120	150	1200	0.65	2.5
	*ρ =* 0.98%					0.98	
	*ρ =* 1.26%					1.26	
	*ρ =* 1.77%					1.77	
Shear span–depth ratio	*λ =* 3	C40	120	150	1200	1.77	2.5
	*λ =* 4				1500		
	*λ =* 5				1800		
	*λ =* 6				2100		
Limb height-to-thickness ratio	*c*_1_/*c*_2_ = 2	C40	120	150	1200	1.77	2.0
	*c*_1_/*c*_2_ = 2.5						2.5
	*c*_1_/*c*_2_ = 3						3.0
	*c*_1_/*c*_2_ = 4						4.0

**Table 6:** Detailed formula of design methods of punching shear capacity.

Design Model	Punching Shear Equation (*V*_c_)
GB50010-2015	*V*_c_ *= v*_c_*u*_0_*h*_0_; $v_{c}=0.7\beta_{h}f_{t}\eta$ ; *β*_h_ is section height influence factor, for *β*_h_ ≤ 800 mm, *β*_h_ = 1.0, for *β*_h_ > 2000 mm, *β*_h_ = 0.8, with linear interpolation used for intermediate values; *f*_t_ is concrete axial tensile strength; *u*_0_ is critical perimeter; *h*_0_ is effective slab thickness; *η* is size effect and section shape influence factor.
ACI 318-19 [27]	*V*_c_ *= v*_c_*u*_0_*h*_0_; $v_{c}=\min\left\{\begin{array}{l} 0.33\lambda \sqrt{f_{c}^{\prime }}\\ 0.17\times \left(1+2/\beta \right)\lambda \sqrt{f_{c}^{\prime }}\\ 0.083\times \left(2+\alpha_{s}\times d/b_{0}\right)\lambda \sqrt{f_{c}^{\prime }} \end{array}\right.$ ; *f*′_c_ is concrete cylinder compressive strength; *β* is aspect ratio of the column; *λ* is lightweight concrete modification factor; *α*_s_ is column position constant, with values 40 for middle columns, 30 for edge columns, 20 for corner columns.
Eurocode 2 (EC2 2004)	*V*_c_ *= v*_c_*u*_0_*h*_0_; $v_{c}=\max\left\{\begin{array}{l} C_{\mathrm{Rd},c}k{\left(100\rho_{l}f_{c}^{\prime }\right)}^{1/3}\\ 0.035k^{3/2}{\left(f_{c}^{\prime }\right)}^{1/2} \end{array}\right.$ ; *C*_Rd,C_ = 0.18; *k* is size effect factor, *k =* 1 + (200/d)^1/2^ ≤ 2.0; *f*_ck_ is concrete cylinder compressive strength; *ρ_l_* is longitudinal reinforcement ratio, *ρ_l =_* (*ρ_l_*_x_*ρ_l_*_y_)^1/2^ ≤ 0.02.

**Table 7:** Critical perimeter assumption of the cross-shaped column–slab connection.

Design Codes	GB50010-2015	ACI 318	Eurocode 2 (EC2 2004)
Square column	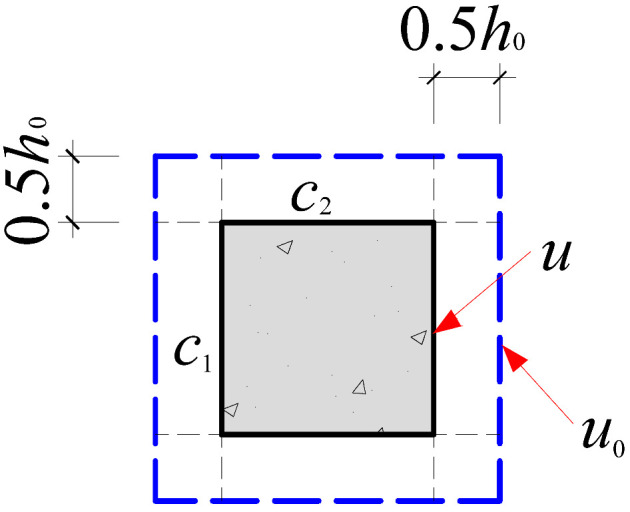	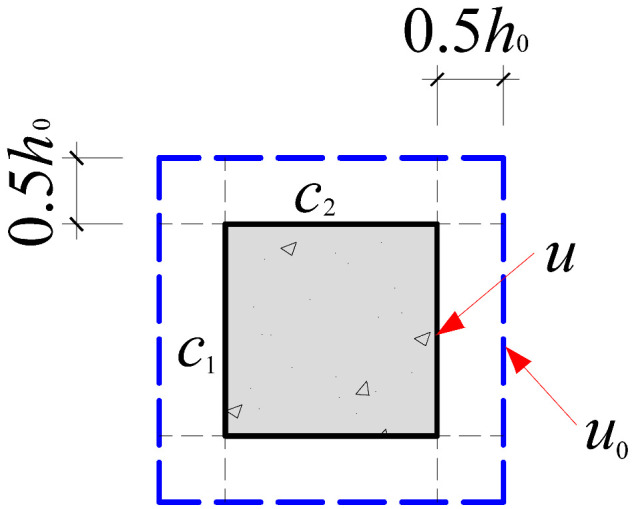	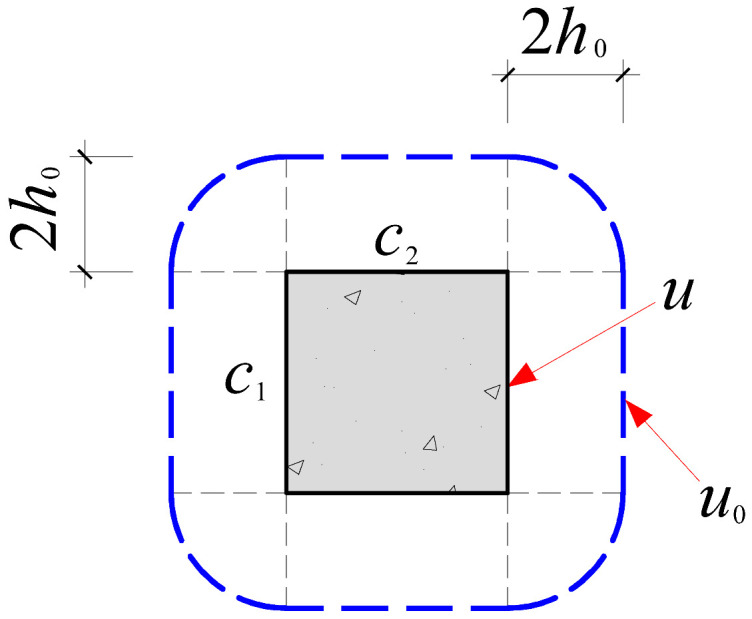
Critical perimeter	*u*_0_ = 2(*c*_1_ + *c*_2_) + 4*h*_0_	*u*_0_ = 2(*c*_1_ + *c*_2_) + 4*h*_0_	*u*_0_ = 2(*c*_1_ + *c*_2_) + 4π*h*_0_
Cross-shaped column	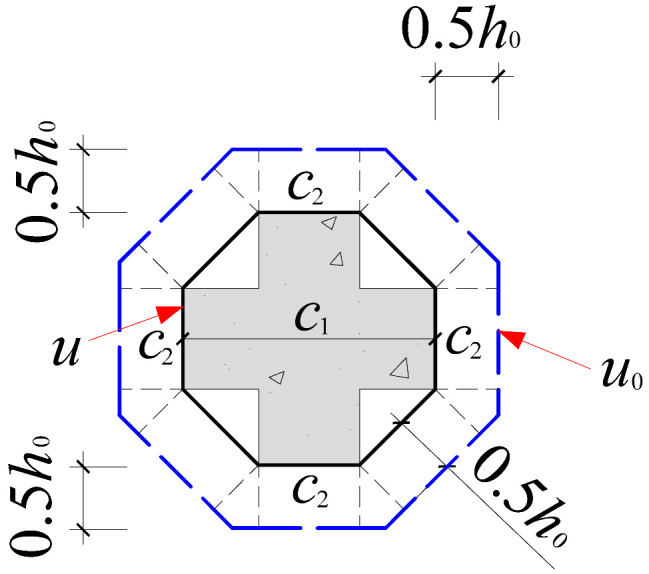	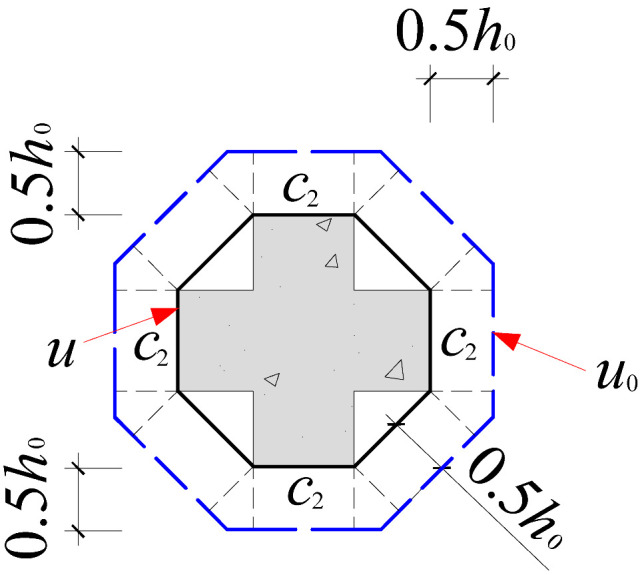	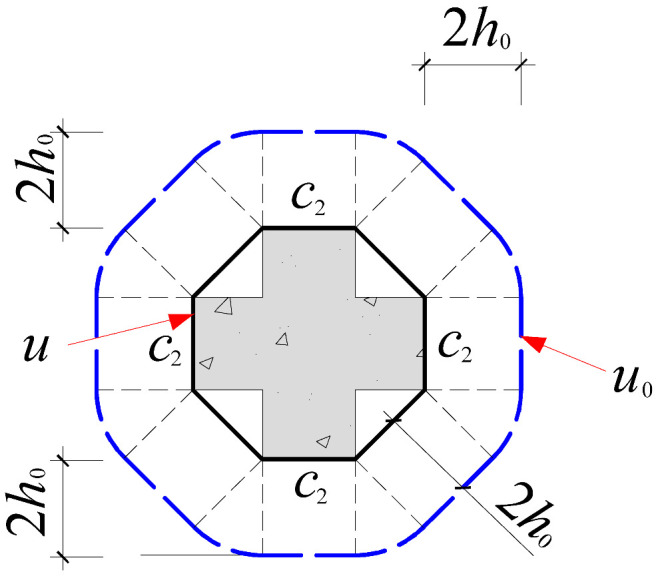
Critical perimeter	$u_{0}=4c_{2}+2\sqrt{2}(c_{1}-c_{2})+8h_{0}(\sqrt{2}-1)$	$u_{0}=4c_{2}+2\sqrt{2}(c_{1}-c_{2})+8h_{0}(\sqrt{2}-1)$	$u_{0}=4c_{2}+2\sqrt{2}(c_{1}-c_{2})+4\pi h_{0}$

**Table 8:** Prediction of punching shear capacity of slab with cross-shaped column–slab connections by design codes.

Specimen No.	Section Dimensions		Slab Thickness	Compressive Strength	*V*_Test_/*V*_FEA_	*V*_Test_/*V*_GB_	*V*_Test_/*V*_ACI_	*V*_Test_/*V*_EC2_
	*c*_1_/mm	*c*_2_/mm	*h*_0_/mm	*f*′_c_/Mpa				
CR-1	300	120	95	26.4	0.98	1.84	1.97	1.54
CR-2	300	120	125	26.4	0.99	1.89	2.02	1.45
				Mean	0.99	1.86	2.00	1.50
				SD	0.01	0.03	0.04	0.06
				COV%	0.01	0.02	0.02	0.04

## Data Availability

The original contributions presented in this study are included in the article. Further inquiries can be directed to the corresponding author.
